# Aboriginal-mainstream partnerships: exploring the challenges and enhancers of a collaborative service arrangement for Aboriginal clients with substance use issues

**DOI:** 10.1186/1472-6963-13-12

**Published:** 2013-01-10

**Authors:** Kate P Taylor, Dawn Bessarab, Lorna Hunter, Sandra C Thompson

**Affiliations:** 1 Curtin Health Innovation Research Institute, Curtin University, Perth, Western Australia, Australia; 2 Combined Universities Centre for Rural Health, University of Western Australia, Geraldton, Western Australia, Australia; 3 Aboriginal Alcohol and Drug Service, East Perth, Western Australia, Australia

**Keywords:** Aboriginal, Mainstream, Vulnerable populations, Partnerships, Health services

## Abstract

**Background:**

Partnerships between different health services are integral to addressing the complex health needs of vulnerable populations. In Australia, partnerships between Aboriginal^1^ community controlled and mainstream services can extend health care options and improve the cultural safety of services. However, although government funding supports such collaborations, many factors can cause these arrangements to be tenuous, impacting the quality of health care received. Research was undertaken to explore the challenges and enhancers of a government initiated service partnership between an Aboriginal Community Controlled alcohol and drug service and three mainstream alcohol rehabilitation and support services.

**Methods:**

Sixteen staff including senior managers (n=5), clinical team leaders (n=5) and counsellors (n=6) from the four services were purposively recruited and interviewed. Interviews were semi-structured and explored staff experience of the partnership including the client intake and referral process, shared client care, inter-service communication and ways of working.

**Results & discussion:**

Communication issues, partner unfamiliarity, ‘mainstreaming’ of Aboriginal funding, divergent views regarding staff competencies, client referral issues, staff turnover and different ways of working emerged as issues, emphasizing the challenges of working with a population with complex issues in a persistent climate of limited resourcing. Factors enhancing the partnership included adding a richness and diversity to treatment possibilities and opportunities to explore different, more culturally appropriate ways of working.

**Conclusion:**

While the literature strongly advises partnerships be suitably mature before commencing service delivery, the reality of funding cycles may require partnerships become operational before relationships are adequately consolidated. Allowing sufficient time and funding for both the operation and relational aspects of a partnership is critical, with support for partners to regularly meet and workshop arrangements. Documentation that makes clear and embeds working arrangements between partners is important to ameliorate many of the issues that can arise. Given the historical undercurrents, flexible approaches are required to focus on strengths that contribute to progress, even if incremental, rather than on weaknesses which can undermine efforts. This research offers important lessons to assist other services collaborating in post-colonial settings to offer treatment pathways for vulnerable populations.

## Background

The complex health needs of vulnerable populations often require input from a variety of health services. In Australia, partnerships between mainstream and Aboriginal services are critical to enhancing service capacity to respond to the complexity of poor Aboriginal health [1,2]. While such partnerships can improve the cultural appropriateness of treatment programs, the legacy of Australia’s history, continuing Aboriginal health disparities and different ways of working can also cause these partnerships to be difficult and sometimes tenuous. With a paucity of research on how to build effective Aboriginal-mainstream partnerships [3,4], it is critical that knowledge is built around how to enhance such partnerships to improve health care delivery.

**Table 1:** Characteristics of research participants

**Organisation**	**Type**	**Services offered**	**Number of Aboriginal staff**
**ACO**	Outpatient AOD service	Urban based AOD counselling & community outreach programs for Aboriginal community members	16
**M1**	Residential Rehabilitation	Inpatient residential AOD treatment in a rural facility modelled on a Therapeutic community approach for clients across cultures	2
**M2**	Residential Rehabilitation	Inpatient residential AOD treatment in a rural facility modelled on a Therapeutic community approach for clients across cultures	2
**M3**	Detoxification Unit	Urban based inpatient AOD medically supported detoxification unit	2

**Table 2:** Participant experiences/perceptions of challenges in the partnership

**Mainstream services**	**Theme**	**Aboriginal organisation**
**■■■■**	Limited knowledge of partner service	**■■■■■**
**■■■■**	Communication challenges	**■■■**
**■■■**	Mistrust and tension	**■■■■■**
**■■■**	Different ways of working	**■■■■■**
**■■■**	Referral / transfer issues	**■■■■**
**■■■**	Resource limitations	**■■■■**
**■■**	Aboriginal staff clinical skills	**■■■**
**■■**	Shared care role confusion	**■■■■**
**■■**	Unequal token partnership	**■■■■**
**■■**	Staff Changeovers	**■■**
**■■**	Personality clashes	
**■**	Politics and Sensitivities	

Strength of response.

**■■■■■** All participants.

**■■■■** Very high.

**■■■** Medium.

**■■** Low.

**■** Very low.

No response.

The National Drug Strategy Aboriginal and Torres Strait Islander People’s Complementary Action Plan 2003-2009 encourages alcohol and other drug (AOD) services to collaborate to broaden programs for the Aboriginal population [5]. In an urban setting in Western Australia, a formal partnership was initiated following a state government decision to redirect funding from an Aboriginal community organisation (ACO) that provided counselling and advocacy to clients with substance issues, to purchase ‘dedicated’ Aboriginal beds at two mainstream inpatient rehabilitation facilities. Clients were to be assessed at the ACO, referred (where necessary) to a mainstream detoxification centre and then transferred to either of two residential rehabilitation facilities for intensive treatment. On completion of treatment in rehabilitation, clients would be encouraged to return to the ACO for aftercare.

With no Aboriginal specific rehabilitation service in the region, one of the goals of the arrangement was to broaden treatment options for Aboriginal clients with AOD issues. The decision was made by the government sector to redirect funding to mainstream agencies with rehabilitation services, requiring them to have ‘Aboriginal beds’ and to employ Aboriginal staff. This paper reports on research conducted with the services involved in the partnership to explore the challenges they faced as well as the factors that strengthened the arrangement.

## Methods

Ethical approval was obtained from the Western Australian Aboriginal Health Information and Ethics Committee (WAAHIEC) and Human Research Ethics Committee of Curtin University prior to data collection.

A team comprising two female non-Aboriginal and one senior female Aboriginal researcher conducted the study. Qualitative interviews with staff (n=16) from the four services engaged in the partnership were undertaken by the two non-Aboriginal researchers who were based within the ACO. Senior managers (n=5), clinical team leaders (n=5) and counsellors (n=6) were purposively recruited in person, via email or telephone, and invited to participate in semi-structured interviews (Table 1). Questions were designed to explore their personal experience and analysis of the partnership including the process of client referrals, shared care, inter-agency communication, partner knowledge and ways of working.

Following explanation of the study, formal written consent was obtained from participants prior to their involvement. Interviews were recorded at the participant’s workplace and transcribed verbatim. Data were analysed thematically using open coding which involved breaking down the data into distinct units of meaning, or codes, according to participants’ responses. After analysis, the 12 most commonly discussed themes were placed centrally in a table, with a column either side for ACO or mainstream responses (Table 2). A notation was then made against each theme if it had emerged during an interview. Where individual participants discussed the same theme multiple times, only one notation was made per interview. In order to adjust for the imbalance in participant representatives from mainstream services (n=12) compared to the ACO (n=4), the number of notations per theme were divided by the total number of participants from either mainstream or the ACO. Themes were then listed in order from highest to lowest to indicate the strength of the response, based on the totalled notations, for each particular theme to give some estimation of the consistency of the recurrence of that theme. Observational data was also recorded by the three researchers in reflective diaries to assist making meaning of the complex interactions taking place. The senior Aboriginal researcher, proficient at working in cross-cultural contexts, supported the research process and analysis from an Aboriginal perspective.

Following data collection and analysis, partnership forums were held at which the issues raised were surfaced and discussed, with the intention of overcoming some of the issues the partnership was experiencing. The senior Aboriginal researcher led this process, using facilitation skills based upon her own experience working effectively in cross-cultural contexts.

## Results & discussion

The interviews revealed a number of issues impacting the quality of partnership, with direct implications on health service delivery. Some of the challenges were related to structural and historical issues while others were attributable to cultural differences. Equally important were the different personalities and stakeholder agendas involved.

### Involuntary partnerships, the redirection of funding & mistrust

Very early in the process of establishing the research, it became clear that the arrangement to redirect funding from the ACO to mainstream services had occurred without freely given agreement with the ACO. It created tension from the beginning which impacted the partnership throughout. The trust of those working for the ACO was effectively undermined by this process due to the coercion that led to the arrangement. This experience caused those in the ACO to question government integrity for having rhetoric and policies espousing support for local Aboriginal-led solutions while enacting a seemingly contradictory practice. The government agency, however, could justify its actions in redirecting these funds in terms of service performance and needs.

The imposed arrangement by which the partnership was established was an ongoing concern for the ACO’s staff and subsequently influenced partnership interactions. Although original partnership arrangements identified direct involvement by the ACO in client intake assessments to the mainstream rehabilitation centres, the final decision was ultimately made by the rehabilitation services with the ACO staff having little control in the process. The lack of standardized documentation clarifying the roles and responsibilities of partners in shared client care and the different expectations of each service complicated partner exchanges. Protracted discussions regarding the ‘ownership’ and management of the “Aboriginal beds” triggered suspicion within the staff and the management of the ACO who saw and reported experiencing the partnership as the redirection of limited Aboriginal organisational funds into mainstream services. From the ACO perspective, although mainstream partners were not directly responsible for the redirection of funding, by receiving it they were complicit in replicating a colonial practice that (once again) undermined Aboriginal self-control.

Although mainstream participants were sensitive to this issue, they also felt frustrated in attempts to communicate and progress the relationship with the ACO, as expressed by this worker:

*the lack of trust that people (at the ACO) might have working with mainstream services - and I understand this for Aboriginal people who have worked in services where there has been this tokenistic offering of funds…but I think when that when that lack of trust becomes a default stance it gets really difficult…*[M2]

The origins of the partnership created a highly contested space that meant it was necessary to revisit it repeatedly in meetings/workshops in order to allow productive exchanges to occur and the partnership to progress.

### Limited knowledge of partner’s services, staff turnover and communication difficulties

Limited knowledge of the other’s services was the most widely reported issue (Table 2). Lack of regular meetings and networking opportunities coupled with high staff turnover in all agencies (a challenging issue often present in health and social services) caused limited knowledge within the Aboriginal and mainstream agencies about each other’s programs. Mainstream agencies also felt this issue contributed to inappropriate and low numbers of referrals. The physical distance between services was also a deterrent to staff from visiting partner organisations and building relationships. Divergent views on communication processes perpetuated this distance. For instance, while maintaining contact was raised as a difficulty for mainstream providers who tended to rely on emails and telephone contact, for the ACO, face-to-face contact was preferred and seen by staff as an important component of building relationships and the different way they did business.

### Divergent views regarding staff skills and competencies

Deliberations around clinical skills in terms of assessing clients for entry into rehabilitation highlighted differences between Aboriginal and mainstream regarding staff competencies. While the ACO prioritised community and cultural knowledge as key staff competencies, for mainstream providers, formal clinical training was given precedence. Knowledge of these different criteria for assessing staff competencies resulted in some ACO staff feeling their mainstream partners perceived the ACO skills as clinically inferior. ACO staff also felt their mainstream partners had little recognition of the complex preparation work they had to do to get an Aboriginal client ready for rehabilitation as articulated below:

*they won’t even let us do the assessment (for client entry into rehabilitation). They think we’re not skilled enough. I think the community or the Aboriginal culture, the Aboriginal knowledge and whatever connections to the community are disregarded. You can go and get all the degrees that you like, you can work with blackfellas, but unless you are a blackfella yourself, you don’t know* [ACO]*.*

While insufficient recognition by mainstream of the skills and working capacity of the ACO was a constant issue for the ACO staff, for some of the rehabilitation workers the lack of clarity around what constituted clinical skills was a major issue. The differences between cultural and clinical competencies and the role that each played in assessment of clients was not clearly identified or articulated in partner documentation, leaving working relationships vulnerable to misunderstandings. For mainstream workers, their attempts to work with the ACO to negotiate these competency based issues proved difficult:

*(our staff) were worried about offending some of the (ACO workers)… there was difference of opinion regarding what constituted clinical skills.. See, there is a context to all of this where people are concerned about offending due to cultural reasons or whatever*…[M1]

Previous studies suggest that limited understanding by non-Aboriginal practitioners of the practical skills that Aboriginal staff need to engage Aboriginal clients in health programs can result in non-Aboriginal co-workers lacking confidence in their Aboriginal colleagues [6-8]. In this partnership, although the partner services had different views of the skill sets required by staff, poor exposure to each other’s work practice exacerbated uncertainty about the clinical competencies of the Aboriginal staff in certain situations. The confidence of the ACO and mainstream services in each other’s programs was undermined by the different perceptions and understandings each had of the other. These tensions fed a mistrust that polarised the partnership.

### Client complexity, resource limits, cultural perspectives and different ways of working

Difficulties were also experienced in the client referral process. Mainstream participants voiced concerns that the ACO were attempting to refer clients too early due to the ‘crisis state’ in which clients were presenting:

…*driven by the referrer because they’re not quite sure what else to do with that person, it’s a desperate situation, you know, homeless. And there is no motivation there (to do a detox program)…*[M3]

However, for the ACO this ‘crisis state’ was not unique – rather, the majority of clients typically presented in a state of crisis, accessing the service spontaneously. For the ACO staff, based on their understanding and experience, not responding to Aboriginal clients immediately could mean ‘*you lose them’.* Also, the ACO staff felt that the different ways they worked with their clients, which combined their cultural knowledge, community obligations and knowledge of the drug and alcohol issues presented, were not fully understood by their mainstream partners - or from the government funders’ perspective. The ACO employees also expressed the view that the complex needs of the client group meant the model of referring clients on to other services for specialised care created major barriers:

*People drop through the cracks. It’s easier to go back home and stick with the drugs and alcohol rather than go on a referral through all these agencies* … *it’s a white fella way* [ACO].

A major unresolved issue between partners concerned different views and organisational culture regarding assessment processes and the issues associated with client referral. The ACO staff felt that culturally appropriate and improved clinical care should involve clients being referred to the ACO first:

*Traditional Aboriginal people will not leave their country, because there are issues around spiritual forces … so the only way we can get (Aboriginal people from outside of the local area) in is to welcome into country … It’s an important part of obligation and responsibility in someone else’s country … we want to run a service on a case management rather than refer-on model, where people have to deal with a number of different agencies. We wanted to stay involved with clients right the way through including when they come out of rehabilitation….* [ACO]

ACO staff believed that relationships and trust were vital for Aboriginal AOD clients and that by having continuity with one service involved in the client’s health care, they would be less likely to fall through referral cracks. The ACO also felt that this model of an Aboriginal service having continuing involvement in the client’s journey could reduce the number of situations when distressed clients in mainstream treatment required intervention to prevent them exiting a program early. However, this ACO-preferred model clashed with mainstream perspectives on the rights of a client to choose what services they are involved in:

*I guess at a philosophical level that goes against everything we believe in terms of empowerment and client choice …You can’t (just) say well this service will meet your needs because you’re Aboriginal. You know, you’re a complex person*…[M2]

The different understanding of client choice was also reflected in the mainstream view that Aboriginal clients do not all respond to treatment in the same way. For example, while group therapy in the therapeutic community (TC) was often debated as to its cultural appropriateness for Aboriginal clients, mainstream providers reported that some Aboriginal clients are not shy and respond well to this approach - highlighting the issue of diversity both in Aboriginal people and the TC setting. While there may be Aboriginal clients who are bi-culturally competent and able to engage with the group therapy approach, there are also Aboriginal clients who struggle with the dynamic, intense and confrontational nature of group therapy. These needs have been iterated in Brady’s comments on Aboriginal residential AOD services: “to be successful, treatment programs should assess the variety of client needs and provide services to meet those needs effectively” [4,9].

The rehabilitation service providers also struggled to understand why staff of the ACO proposed their involvement in the care of all Aboriginal clients from the onset, even if the client was being referred from another service in a rural or regional area. From the ACO staff’s perspective, being able to welcome an Aboriginal person from a different language group to their country constituted an Aboriginal protocol that was also seen as an integral and inclusive part of their healing process. ACO staff reported their experience of often being called at crisis point by the mainstream organisations that did not include them in the front end of the admission to intervene in a client’s treatment and provide cultural support to prevent the client from leaving rehabilitation. Thus, it was not that clients did not have the freedom to choose, but rather ACO staff were trying to articulate the importance of adhering to cultural protocols and establishing a relationship as an integral component to the client’s health care journey. Identifying what cultural appropriateness actually means in practice and in terms of the diversity of Aboriginal experience, and how this intersects with mainstream values and service based requirements created tensions in the partnership. While treatment options were theoretically broadened through the partnership, poor understanding of the ways the different organisations worked, the philosophical positions informing their practice and the complexity of issues inherent to the client group, in fact complicated the treatment pathway in practice.

### Client behaviour and colonial footprints

Historically-linked issues pertaining to Aboriginal-mainstream relations also complicated how the partners considered the shared care of clients. For example, some mainstream staff felt on occasion, ACO staff ‘used’ the negative experiences of Australia’s colonial history to explain client behaviour and justify the need for different treatment approaches. For these mainstream participants, while the wrong-doings of the colonial past were acknowledged, they felt it should not be used as an excuse for certain unfavourable client behaviours (such as violence or anger) in treatment. Deliberations with ACO staff were complex and sensitive, and mainstream staff commented that their non-Aboriginality was often used unreasonably against them by ACO staff whom suggested that they could not understand an Aboriginal client’s situation because they were not Aboriginal.

The dilemmas around what constituted acceptable and unacceptable behaviours of clients during AOD treatment was a contentious issue between the ACO and mainstream. While ACO staff attributed some of the client behaviours to the trauma of Stolen Generation (a term referring to children of Aboriginal and Torres Strait Islander decent who were forcibly removed from their families by Australian government policies until the late 1960’s) and experiences of racism, mainstream services saw them as controlling, adaptive and coping behaviours attributable to the AOD pattern of addiction. As part of a duty of care to the flexible and changing TC environment, some clients were not ready for rehabilitation and needed to work on anger issues before admission- a problematic position for ACO staff who felt some Aboriginal clients needed to be *admitted* immediately but *managed* differently. Acknowledging and working with injustices which stemmed from the past while attempting to address the AOD addiction needs of the present raised ongoing issues for both mainstream and Aboriginal partners in providing a united level of care for Aboriginal clients.

### Organisational, cultural and personal tiers of influence

When conflict arose in the partnership, considerable difficulties were experienced in terms of locating the ‘cause’ of the issue within the matrix of colonial experiences, cultural values, organisational interests and individual personalities. One participant reflected:

*You’ve got people taking organisational positions…and there’s a lot of emotional, personal stuff happening …. So that makes it hard to extract the cultural stuff… when you get the response you never know what it’s about, you never know what you’ve triggered off…unless we are actually able to put them on the table then we are never able to understand well what’s a cultural issue and what’s an organisational one* [M1]

These complexities were further challenged by a lack of regular meetings and communication. There was a considerable influence of individual personalities and their leadership and communication style which impacted on partner engagement. Governance arrangements meant the influence of positions (in terms of positional authority) and individual personalities were perceived as often overpowering the capacity of a service to collaborate, and reinforced the sense that not everyone was committed to the partnership.

Other concerns related to their experience of anger being directed at non-Aboriginal staff by staff in the ACO and their perception that being Aboriginal was being used to justify having a *‘right to be angry’*:

*Staff … felt apprehensive about giving (the ACO staff) feedback about certain clients who they thought were not suitable … or had to discharge them or discipline them… at times it has affected their decision making in relation to the client* [M1].

Mainstream partners also expressed concern that there was an agenda within the ACO to have its own rehabilitation facility (and hence a lack of commitment to make the pathways provided through the partnership work effectively). Another factor that pressured the partnership was the marginalisation felt by the ACO in the AOD sector, as the only Aboriginal service in the partnership and committed to working in a way that would suit their clients. Early in the project, these different issues resulted in both ACO and mainstream staff often feeling meetings were unproductive as underlying concerns were not openly discussed.

### Enhancers

Despite the issues the partnership was encountering, staff in all services were positive about the opportunities created through the arrangement in terms of widening the range of treatment options for Aboriginal clients. The ACO staff felt the partnership created a chance for its staff to learn different ways of working with Aboriginal clients with AOD issues, while mainstream providers felt they were learning how to tailor their service and treatment techniques to a more culturally inclusive approach. Importantly, the ACO also discussed the flow on effect of mainstream and Aboriginal services working together on clients’ healing:

*If those two agencies are working together, it will make a big difference …(to the clients). They can trust … then they believe in themselves a bit more. There’s a lot more Aboriginal presence in the AOD sector generally. Word gets out that they do feel OK there.* [ACO worker].

Mainstream staff also spoke about the positive impact of the partnership as exposing non-Aboriginal clients and staff to Aboriginal people, enriching the therapeutic community setting, building relationships across socio-cultural groups and thus more broadly supporting reconciliation.

### Implications

This study explores an example of a partnership developed involuntarily (and under coercion from some Aboriginal people’s perspective) as funding was moved away from one service to another. The partnership was thus imposed in the absence of trusting relationships between services, despite previous criticism of mainstream processes pushing collaborations with Aboriginal partners before trust has adequately matured [7]. Successful Aboriginal-mainstream partnerships demonstrate the importance of building arrangements based on genuine trust [10][11-14]. Given the increasing focus on requiring inter-service collaborations, funders need to understand and act on the knowledge that the human and financial cost implications of a dysfunctional arrangement due to an immature partnership may be far greater than the initial outlay of resources devoted to building a collaboration based on mutual trust, equity, benefit and genuine interest.

Previous studies have identified partner uncertainty as a key source of tension [6,7]. Findings from this study reiterate the importance of clarifying roles and expectations and ensuring regular opportunities for staff exchanges and networking are created so partners can build understanding of one another’s skills, ways of working, and strengths. Committing to regular partnership workshops proved very helpful in resolving tensions, while the use of a skilled, bi-culturally competent facilitator able to create a safe space for discussing difficult issues was critical to making such sessions productive. Further, while adequate funding to realistically support the activities of the services is important [13], committing funds to support services to come together regularly and workshop their partnership is also invaluable. Ineffective resource planning leading to an unsuccessful partnership can compound past government and mainstream failures in addressing Aboriginal health, angering those Aboriginal people directly involved and their communities [15].

Developing standardized documentation to clarify service roles helped to ameliorate some of the issues associated with staff turnover. Such documents should recognize contractual arrangements and equity, as well as the process for sharing client information and care and practical aspects of managing the relationship. Using tools to support partnership processes is also suggested. For example, partners could consider co-developing measures of effectiveness to monitor and assess relational factors (e.g. communication, vision); operational factors (e.g. ease of referral-admissions, regular scheduled meetings) and client outcomes (e.g. length of treatment, service engagement by clients, client feedback). Such assessments would also support the definition of responsibilities, shared duty of care and fostering of culturally secure and transparent practices. Aligning such support tools with continuous quality improvement processes could also assist accountability and funders to recognise the effort and activity involved in maintaining an effective, cross-cultural inter-service partnership.

Inevitably, the style of engagement and approach to practice will reflect the individual personalities within a particular service. In terms of a collaborative arrangement, the relationships between key players at both the senior management and grassroots level have the capacity to either actively champion what is captured in standardized documents or render it impotent. This is why key indicators for success from other Aboriginal-mainstream partnerships illustrate the importance of recognizing that tensions will arise and having effective mechanisms to work to resolve them [6,7,14]. Talking early, across the many layers within the services (from senior managerial to clinical practice level) will create greater room for issues to be explored and individual personalities to engage. A successful partnership will be strongly dependent on the practical strategies services develop to remain attentive to the core cross-cultural and individual relational process - with benefits ultimately flowing on to enhanced client outcomes.

While Aboriginal-mainstream partnerships offer an important step in addressing the underlying determinants of Aboriginal health [6,7,16,17], to be successful these partnerships must be sympathetic to the pressures facing Aboriginal organisations in meeting mainstream demands whilst maintaining strong cultural and community roots. Aboriginal structures of organisational governance often bear the weight of operating between these two organising systems [18], and partnerships can face considerable difficulties if these pressures are not clearly understood [19]. The ‘costs’ for Aboriginal organisations in staying connected and engaged with the community is not always measurable or captured in activity reports. Identifying what accountability and activities look like from an Aboriginal community perspective is clearly needed. While responsibility and accountability are important notions, there must be recognition of how much is added to their service to Aboriginal clients by the involvement of Aboriginal staff-and their different ways of working. Aboriginal interpretations of what a partnership model looks like in practice are a critical contribution [20]. Importantly, Aboriginal organisations must be proactive in defining reasonable guidelines for engagement, illustrated through organisation policies outlining partnership terms and conditions, to assist laying foundations of inter-service collaborations that will meet their values, interests and concerns.

## Conclusion

While partnerships between Aboriginal and mainstream organisations can often face challenges, for the most part, the enormous potential of these arrangements is widely acknowledged and organisations are generally concerned – albeit to varying degrees - with making them work. This case study highlights the importance of establishing and maintaining, through ongoing human and resource investment, deliberative forums and mechanisms to support partners to recognize tensions early and explore strategies to address them. Facilitators, who are trusted and respected by all partners, capable of operating effectively in the bicultural space and who can actively involve all perspectives without privileging individual voices, are crucial to the success of such forums. Importantly, facilitators need to be equipped to assist partners to hold courageous conversations about the often unspoken difficult issues - the ‘elephants in the room’. Although often premised on contractual agreements, partnerships between services are ultimately built on relationships between people, with regular dialogue and an opportunity to reflect on behaviours crucial to understanding inter-relational issues. Thus, while the context for Aboriginal and mainstream partnerships may often be challenging, partners who commit to regular, ongoing facilitated forums also create space for tending, respecting and improving the relationships between the individuals that exist within them. The use of the reported findings of this research was part of a broader Action Research process using facilitated Partnership Forums. These Forums proved highly transformational, with the process and results to be reported in a subsequent publication.

## Endnotes

^1^ In this paper, the term Aboriginal will be used. This paper was written in Western Australia, where ‘Aboriginal’ is the preferred name by the traditional owners of the land.

## Competing interests

The authors declare there are no competing interests.

## Authors’ contributions

KT undertook the research, analysis and drafted this manuscript. DB had instrumental involvement in the research design, analysis and manuscript draft. LH gave advice and collaborated on the research design and analysis. ST conceived the initial study design and was involved in drafting the manuscript. All authors read and approved the final manuscript.

## Pre-publication history

The pre-publication history for this paper can be accessed here:

http://www.biomedcentral.com/1472-6963/13/12/prepub
